# qpure: A Tool to Estimate Tumor Cellularity from Genome-Wide Single-Nucleotide Polymorphism Profiles

**DOI:** 10.1371/journal.pone.0045835

**Published:** 2012-09-25

**Authors:** Sarah Song, Katia Nones, David Miller, Ivon Harliwong, Karin S. Kassahn, Mark Pinese, Marina Pajic, Anthony J. Gill, Amber L. Johns, Matthew Anderson, Oliver Holmes, Conrad Leonard, Darrin Taylor, Scott Wood, Qinying Xu, Felicity Newell, Mark J. Cowley, Jianmin Wu, Peter Wilson, Lynn Fink, Andrew V. Biankin, Nic Waddell, Sean M. Grimmond, John V. Pearson

**Affiliations:** 1 Queensland Centre for Medical Genomics, Institute for Molecular Bioscience, University of Queensland, St Lucia, Brisbane, Queensland, Australia; 2 The Kinghorn Cancer Centre, Cancer Research Program, Garvan Institute of Medical Research, Darlinghurst, Sydney, New South Wales, Australia; 3 Department of Surgery, Bankstown Hospital, Bankstown, Sydney, New South Wales, Australia; 4 South Western Sydney Clinical School, Faculty of Medicine, University of New South Wales, Liverpool, New South Wales, Australia; 5 The University of Queensland, UQ Centre for Clinical Research, Brisbane, Queensland, Australia; 6 University of Sydney, Sydney, New South Wales, Australia; 7 Cancer Diagnosis Group, Northern Cancer Translational Research Unit, Royal North Shore Hospital, St Leonards, New South Wales, Australia; Cleveland Clinic Foundation, United States of America

## Abstract

Tumour cellularity, the relative proportion of tumour and normal cells in a sample, affects the sensitivity of mutation detection, copy number analysis, cancer gene expression and methylation profiling. Tumour cellularity is traditionally estimated by pathological review of sectioned specimens; however this method is both subjective and prone to error due to heterogeneity within lesions and cellularity differences between the sample viewed during pathological review and tissue used for research purposes. In this paper we describe a statistical model to estimate tumour cellularity from SNP array profiles of paired tumour and normal samples using shifts in SNP allele frequency at regions of loss of heterozygosity (LOH) in the tumour. We also provide qpure, a software implementation of the method. Our experiments showed that there is a medium correlation 0.42 (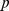-value = 0.0001) between tumor cellularity estimated by qpure and pathology review. Interestingly there is a high correlation 0.87 (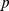-value 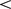 2.2e-16) between cellularity estimates by qpure and deep Ion Torrent sequencing of known somatic *KRAS* mutations; and a weaker correlation 0.32 (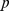-value = 0.004) between IonTorrent sequencing and pathology review. This suggests that qpure may be a more accurate predictor of tumour cellularity than pathology review. qpure can be downloaded from https://sourceforge.net/projects/qpure/.

## Introduction

Solid tumors are comprised of a variety of cell types, including neoplastic cells and cells which make up the stroma (e.g. connective tissue, blood vessels and inflammatory cells). Stromal cell contamination is a key consideration in cancer genome studies as the sensitivity of copy number analysis, mutation detection, cancer methylation and cancer gene expression analysis are all confounded by increasing amounts of normal cells in a tumour [1]–[3]. Accurately estimating the tumor cellularity in genomic samples is therefore an important first step in cancer genome experiments.

Pathology review of specimens is the most common method to estimate tumour cellularity. It is based on the reviewing of tissue sections taken from a tumor specimen. Ideally this is carried out on the same tissue block used for DNA extraction. In many cases, however, the pathological review is carried out on sections well removed from the tissue from which DNA is extracted. In this case, irregularities in tumour shape and heterogeneity in stromal cell contamination can confound cellularity estimates. Alternative approaches to cellularity estimation assay the DNA sample directly.

There are several tools that can directly estimate tumour cellularity from single nucleotide polymorphism (SNP) microarray data. SOMATICs was developed to identify copy number changes in SNP microarray data and reports the percent of the sample which contains each event, this can be used to infer tumour cellularity, however the tool is computationally expensive and works best in samples containing 40–75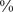 cancer cells [4]. ASCAT was also developed to identify copy number changes, however during this process ASCAT initially estimates the fraction of aberrant or tumour cells in the sample [5]. SiDCon is a spreadsheet based application which can determine the level of stromal contamination [6]. Both these tools were originally developed for SNP microarrays containing thousands of probes and lack scalability to process current SNP microarrays with millions of probes.

Tumor cellularity can also be estimated based on the quantification of mutant alleles by sequencing. This approach requires prior knowledge and careful selection of the mutation to ensure it is an early/driver event in the cancer. In pancreatic cancer the *KRAS* gene is a hotspot for somatic mutations and is frequently mutated [7]. *KRAS* mutations are early events in pancreatic cancer, thus the mutations are thought to exist in all malignant cells. High-throughput pyrosequencing sequencing technology is the more sensitive assay for *KRAS* mutation detection compared to the dideoxy sequencing [8]. Ion Torrent sequencing technology [9] is one of the current pyrosequencing technologies used in our laboratory and provides faster sequencing runs and deeper coverage compared to other approaches [10].

In this study we have developed a tumor cellularity prediction model (qpure), which uses SNP microarray data from paired (tumor and normal) samples to directly estimate tumor cellularity for a given sample. This method has the advantage that the DNA sample used to run the SNP arrays for qpure cellularity determination is the same sample used for future genomic studies such as sequencing. To define the model, DNA was taken from a matched pair of normal tissue and cancer cell line and mixed at predefined ratios to create a set of 14 standards for which the tumour cellularity was known. The qpure method was applied to SNP data from each of these mixtures to create a standard curve against which other samples could be compared. We describe the model and compare the cellularity predictions to pathology estimates and Ion Torrent sequence data and show that the qpure tool can accurately predict tumor cellularity.

## Materials and Methods

### Ethics Statement

Informed consent was obtained in written form from each donor. Ethics approvals were granted in written form by the medical research ethics committee of the University of Queensland (Project Number: 2009000745); the human research ethics committee of Westmead Hospital (Reference Number: JH/JL HREC2002/3/3.19 1402); the human research ethics committee of NSW Health Western Zone (Project Number: 2006/054); the human research ethics committee of NSW Department of Health (Protocol Number: X11-0220 HREC/11/RPAH/329); the HARBOUR human research ethics committee of Northern Sydney Central Coast Health (Protocol Number: 0612-251M); the research ethics committee of Royal Adelaide Hospital (Protocol Number: 091107a); the human research ethics committee of Metro South Health Service District (Reference Number: HREC/09/QPAH/220); the human subjects research institutional review boards of Johns Hopkins (Study Number: NA_00026689); the human research ethics committee of South Metropolitan Area Health Service (Reference Number: 09/324); the St John of God Health Care Ethics Committee (Reference Number: 385); the human research ethics committee of the Southern Adelaide Health Service (Application Number: 167/10); the human research ethics committee of Austin Hospital (Protocol Number: H2011/04083).

We are unable to provide a test data set as all tumor/normal pairs processed under the aegis of the Australian ICGC effort are subject to ICGC data release guidelines. ICGC requires that all genomic data be lodged in public data archives including the ICGC Data Portal (http://dcc.icgc.org/) and the European Genome-phenome Archive (EGA, https://www.ebi.ac.uk/ega/), however, due to ethics and privacy concerns, ICGC requires that the public archives and all participating nations agree that no germline data be made available without the access request being processed through the ICGC Data Access Committee (DACO). Many non-ICGC cancer projects operate under similar data access restrictions and we were unable to identify an equivalent alternative publicly available paired tumor/normal genotype and sequencing dataset.

### DNA Extraction and SNP Microarray Analysis

A total of 5 pancreatic cancer cell lines and 76 pancreatic tumour samples were used in this study (Table S1). DNA was extracted from samples, matched normal tissue and pancreatic cell lines using the AllPrep DNA/RNA kit (Qiagen). 200 ng of each DNA sample was profiled using 1 M HumanOmni-Quad BeadChip (Illumina) following the manufacturers protocol. Chips were scanned using an IScan (Illumina) and the B allele frequency (BAF) and log R ratio (LRR) intensity values for each SNP calculated using the GenomeStudio genotyping module v1.84 (Illumina).

### Model Generation on Mixing Experiment

**Figure 1:**
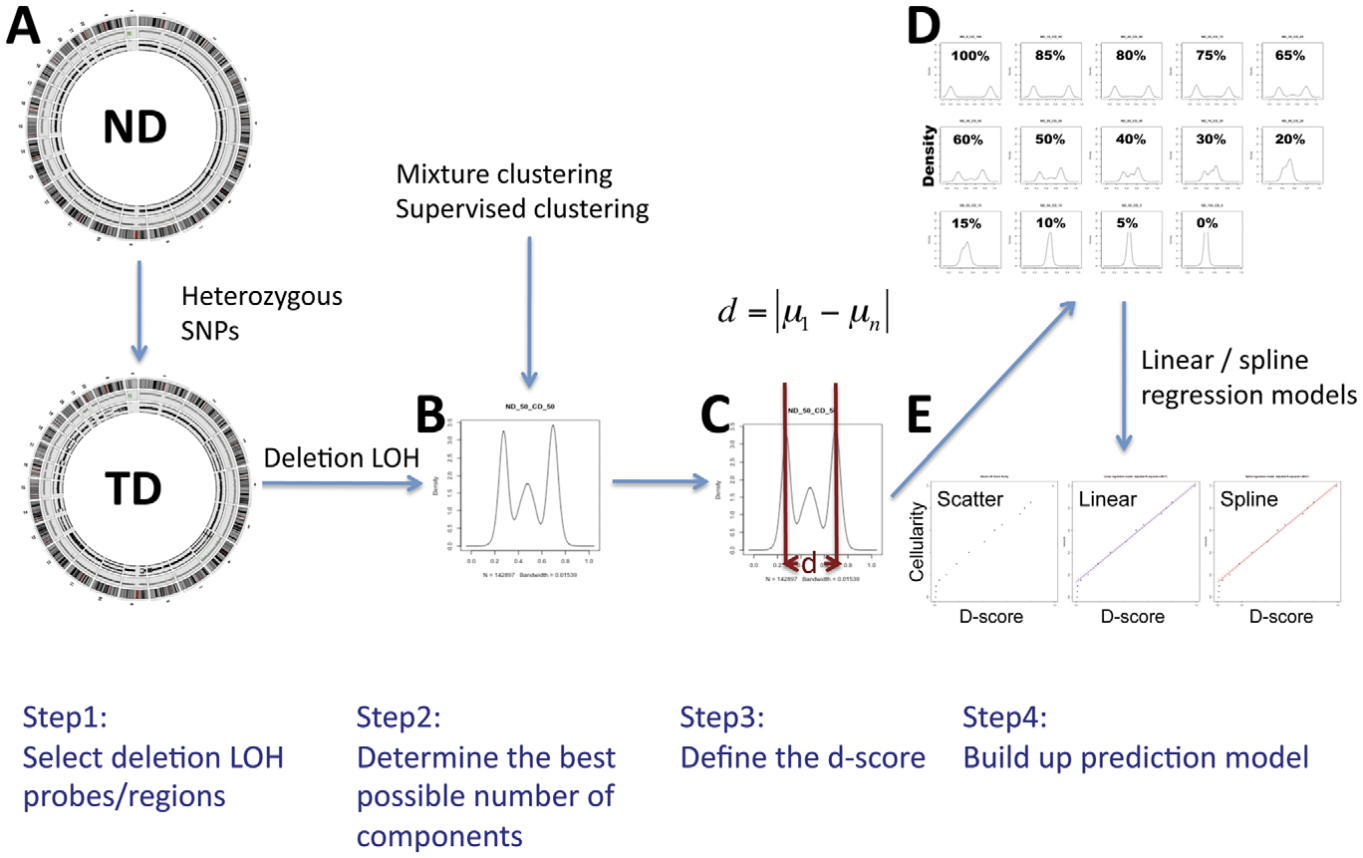
Overview of the qpure method. Circos plots of the SNP array data for a paired normal (ND) and tumor (TD) sample showing regions of LOH in the tumor sample (A). The chromosome ideograms are shown on the outer wheel, the logR and BAF values are plotted in the middle and inner wheel respectively. The density plot of the probes in LOH regions (B) is used to calculate the d-score (C). The d-score is compared to the density plots of probes within regions of LOH for the cell line: normal DNA mixtures which represent different cellularity (D). The d-score and cellularity are highly correlated (E). Three plots from the left to the right are the scatter plot only, with fitting the simple linear model and with fitting the spline regression model respectively.

To create the qpure model a SNP microarray mixture experiment was performed whereby DNA from a cell line and a matched normal DNA sample from the same patient were mixed at 14 predetermined ratios to mimic a broad range of tumour cellularities (Table 1). The qpure cellularity prediction model contains four major steps (Figure 1).

**Table 1:** Design of mixing experiments.

Tumor Cellularity	Sample ID	Mixture
100%	ND_0_CD_100	100% cell line tumor DNA
85%	ND_15_CD_85	85% cell line tumor DNA
80%	ND_20_CD_80	80% cell line tumor DNA
75%	ND_25_CD_75	75% cell line tumor DNA
65%	ND_35_CD_65	65% cell line tumor DNA
60%	ND_40_CD_60	60% cell line tumor DNA
50%	ND_50_CD_50	50% cell line tumor DNA
40%	ND_60_CD_40	40% cell line tumor DNA
30%	ND_70_CD_30	30% cell line tumor DNA
20%	ND_80_CD_20	20% cell line tumor DNA
15%	ND_85_CD_15	15% cell line tumor DNA
10%	ND_90_CD_10	10% cell line tumor DNA
5%	ND_95_CD_5	5% cell line tumor DNA
0%	ND_100_CD_0	0% cell line tumor DNA

#### Step One: Select probes in regions of loss

**Figure 2:**
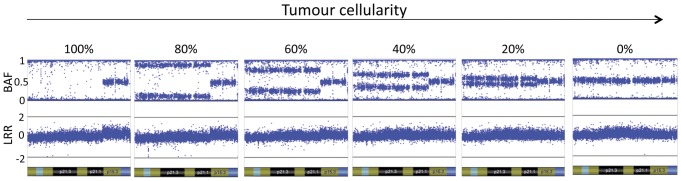
B allele frequency (BAF) and log R ratio (LRR) plots for a region of LOH with changing tumor cellularity. DNA from a cancer cell line and matched normal DNA were mixed in different proportions and assayed using SNP arrays. BAF and LRR plots were generated using GenomeStudio software (Illumina). For illustrative purposes a region of loss on the p arm of chromosome 7 in the cancer cell line is shown. In the 100 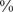 normal sample (0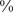 tumor) the SNPs are either heterozygous (BAF 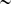 0.5) or homozygous (BAF = 0 or 1). In regions of single chromosome loss in the tumour there is LOH. In the 100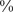 cell line the BAF is showing a homozygous state and there is clear loss in the LRR. As tumour cellularity decreases the separation of the BAF decreases.

To ensure homozygous SNPs in the normal sample do not confound the analysis, heterozygous SNPs from the normal sample were filtered to select those in regions of single-copy loss in the matched tumour sample. These SNPs should all show genotype AB in the normal sample and either A or B in the matched tumour sample. The DNA from any normal cell contamination within the tumour sample reintroduces some of the lost allele and shifts the observed allele frequency back towards genotype AB. The magnitude of the shift is directly related to the proportion of contaminating normal cells (Figure 2). To select SNPs which show deletion of one allele in the tumour a threshold method was employed, whereby a cutoff value was chosen to determine the selection of the SNPs [11]. In the qpure method, the cutoff value was calculated separately for each sample using the median of all the selected SNPs minus the standard deviation of middle 50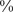 quantile.

#### Step Two: Determine the best possible number of components to describe the distribution of the BAF

The distribution of the BAF for selected SNPs in regions of loss was determined in order to accurately identify the clusters. Two different methods were used: a supervised clustering method k-means clustering and an unsupervised mixture modeling method. For a set of n observations (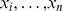) each of which is a 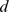-dimensional vector, k-means clustering [12] aims to partition the points into K clusters (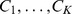) so that the within-cluster dispersion is minimised. It is described as
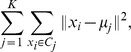
where 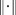 denotes Euclidean distance, and 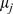 is the center of cluster 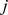 which in our case can be computed as the mean of the 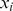 in the cluster.

Unlike the k-means clustering method, the mixture model [13] does not require the number of clusters to be predefined. Using either the Akaike information criterion (AIC) or the Bayesian information criterion (BIC) the model can search for the optimal number of clusters or partitions. For a set of n observations (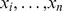) that are assumed to come from a mixture of 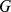 groups in some unknown proportion (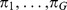) the mixture model is described as
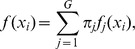
where 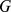 is the best number of clusters or partitions selected based on AIC or BIC criteria, 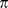 is estimated from the data using the expectation maximization algorithm, and the feature vector 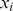 takes the mixture density function 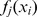 in group 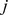. By default the mixture model by Fraley and Raftery [13] estimates the parameters based on the optimal number of clusters in the model as determined by BIC.

#### Step Three: Define the d-score that is related to tumour cellularity

The d-score for each sample is defined as the absolute distance between centers of the two furthest clusters. These clusters represent SNPs that are in regions of LOH in the tumour cells. And the d-score can be computed as
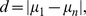
where 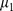 and 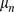 represent the means of the two furthest clusters.

#### Step Four: Modeling the relationship between d-score and tumour cellularity

To derive a model that could be used to predict tumour cellularity from the d-score, both a simple linear model and spline regression model were employed the data from the 14 synthetic samples where the cellularity was known. Given a set of 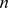 points 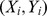, 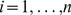 a simple linear regression model can be formulated as
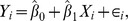
where 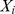 is the d-score (see Step 3) and 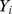 is the cellularity. The spline regression model [14] can be formulated as



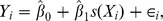
where 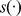 is the smoothing function using penalized regression splines that are designed to be optimal.

### Validation of Different Predictive Models

The leave-one-out cross-validation method was used to validate performance of the different predictive models (Table 2). These predictive models include different combinations of clustering methods and prediction models. The testing score is defined by
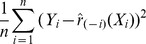
where 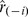 is the cellularity estimation obtained by omitting the 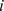
th pair 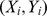.

**Table 2:** The leave-one-out cross-validation results for each model in the qpure method.

No	Model	Prediction Error
1	K-Means + Linear regression	0.5%
2	K-Means + Spline regression	0.3%
3	Mixture clustering (1∶3) + Linear regression	0.2%
4	Mixture clustering (1∶3) + Spline regression	0.16%
5	Mixture clustering (1∶5) + Linear regression	3.4%
6	Mixture clustering (1∶5) + Spline regression	2.8%
7	Mixture clustering (1∶x) + Linear regression	0.2%
8	Mixture clustering (1∶x) + Spline regression	0.13%

In the second column the number in the brackets is the pre-defined number of components. The smaller prediction error is related a better prediction model.

### Cellularity Estimation from Pathology and Deep-sequencing of *KRAS* Mutations

Tumour cellularity was estimated by an anatomical pathologist and sequencing of *KRAS* mutations was performed as an alternate molecular measurement of tumour cellularity. Barcoded primers were designed to amplify *KRAS* exon 2 and 3 (Table S2). These exons are frequently mutated in pancreatic cancer [7] and are known to harbor both driver/founder mutations and represent a hot spot for somatic mutations in pancreatic cancer. Amplicons spanning the highly perturbed codons (9,12,13,59,61) of exons 2 and 3 were generated and products were subsequently pooled and subjected to Ion Torrent sequencing to an average depth of 5218 fold (range 609 to 21770) and 4145 fold (range 102 to 23980) in the tumour and normal samples respectively. Identification of somatic mutations was performed by sequence pileup and the cellularity was calculated by determining the percentage of reads bearing the mutation multiplied by a factor of 2 (assumes the *KRAS* mutation is heterozygous).

### Comparing Cellularity between Pathological Estimations, qpure Estimations, *KRAS* Sequencing and ASCAT Estimations

The correlation between pathological scores, qpure, *KRAS* sequencing and ASCAT estimations was calculated either as a Pearson’s correlation or a Spearman’s rank correlation. For comparing the difference between two or three groups (different estimation of tumour cellularity) either a two-sample t-test or ANOVA test was employed.

## Results

To create the qpure model SNP microarray experiments were performed on a series of normal and cancer cell line DNAs mixed at predetermined ratios to represent different tumour cellularites (Table 1).

### The Relationship between Tumour Cellularity and the Distribution of the BAF within Regions of LOH

In a normal diploid sample, SNPs occur in either a heterozygous or homozygous state. Tumours are characterized by genomic instability that frequently manifests itself as regions of DNA copy number change. Loss of heterozygosity (LOH), or the loss of one copy is a common event and manifests as regions of somatic change of heterozygous SNPs to hemizygous SNPs. The distribution of the BAF of SNPs in regions of LOH varies with the percentage of tumour to normal DNA in the sample (Figure 2). The BAF distribution within regions of LOH can be presented as two peaks which are close to the homozygous state (0 and 1) in samples with high tumour content and which move towards the heterozygous state (0.5) as the tumour content decreases (Figure 1D or Figure S1).

### The Relationship between Tumour Cellularity and the d-score

We created a d-score that measures the absolute distance between the two major BAF peaks and which can be used to predict tumor cellularity. Two models were used to predict tumour cellularity from the d-score: a k-means model and a mixture model. The tumour cellularity is linearly correlated to the d-score when the tumor cellularity is between 20–100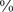, but not at cellularities 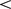
20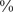 (Figure S2). This might be because the SNP arrays are insensitive for very low cellularity samples or both the k-means and mixture model are underestimating the best components when the distribution is uni-modal for low cellularity samples. Therefore a spline regression model was also implemented for cellularity prediction.

The stability and reliability of the d-score was tested by choosing different log R ratio cut-off values to select probes within regions of loss. Nine cut-off values were tested ranging from 1 percentile to 100 percentile of negative log R ratio values (Figure S3). SNPs with log R ratio values lower than the testing cutoff values were used in the model to estimate d-score and cellularity. The analysis showed that the d-scores changed with the percentage of tumor DNA in the sample, however, changing the threshold (cutoff values) for selecting SNPs in regions of loss did not affect the d-score significantly.

### Validation of Cellularity Prediction Models

A leave-one-out cross-validation method was used to determine the best model for cellularity prediction (Table 2). All prediction models produced a prediction error (PE) of less than 5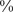 and the mixture model without predefining the number of cluster (1:x) with spline regression performed the best (PE = 0.0013). The spline regression models perform best as they not only describe the linear relationship between d-score and the amount of tumour DNA above 20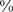, but also allow the model to adjust for samples with lower amounts of tumor DNA using the spline curve. Consequently the qpure tool has been developed allowing for all models to be used, however the mixture-clustering model combined with spline regression is the default model used for cellularity prediction.

To further validate qpure, the model was used to estimate the tumour cellularity, from SNP microarray data, of 5 pancreatic cell lines, as cell lines are considered to be free of normal cell contamination. The cellularity of the five pancreatic cell lines (Table S1) were predicted as 99.8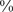, 100.0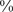, 99.5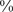, 100.0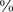 and 99.9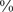.

### Cellularity Estimation in Pancreatic Primary Tumours

**Figure 3:**
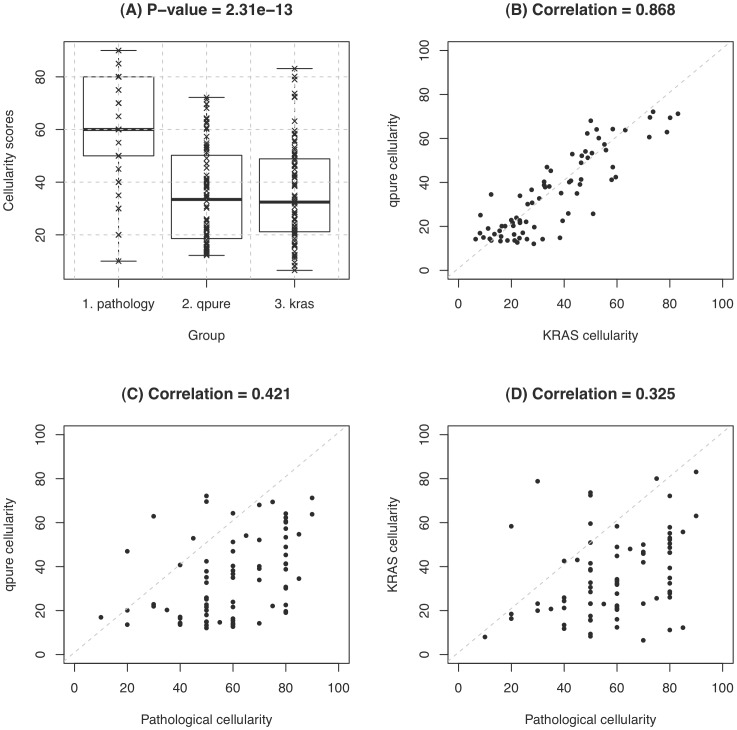
Correlations of cellularity estimated by different methods in a pancreatic cancer cohort. Cellularity was predicted in the pancreatic cohort using 3 methods: pathology review, qpure and deep Ion Torrent sequencing of *KRAS*. Cellularity predictions are shown in the boxplot (A), the 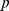-value was calculated using an ANOVA test to determine whether on average there is difference between the cellularity scores returned by the different methods. The correlation between each method using Spearman’s rank correlation was calculated (B–D). Scatter plots are shown which compare *KRAS* deep sequencing and qpure estimates (B), qpure and pathology estimates (C), and *KRAS* deep sequencing and pathology estimates (D).

DNA from a cohort of 76 primary pancreatic adenocarcinomas was assayed using SNP microarrays and the qpure tool was used to predict sample cellularity. The tumour cohort was also subjected to pathological review where the sections for review were taken from the surface of the fresh frozen tissue blocks used to isolate tumour DNAs. Cellularity was also predicted for those tumours bearing heterozygous *KRAS* mutations after deep *KRAS* sequencing (Table S3). The pathology, *KRAS* sequencing and qpure cellularity estimates ranged from 10 to 90 percent (59
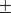
18), 7 to 83 percent (36
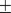
19) and 12 to 72 percent (35
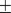
18), respectively. *KRAS* deep sequencing and qpure estimates showed the closest concordance (Figure 3A), with a correlation of 0.868 (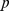-value 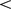 2.2e-16) (Figure 3B). Both qpure and deep sequencing cellularity estimates were only moderately correlated to the histological estimates: 0.421 (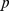-value = 0.0001) and 0.325 (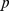-value = 0.004) respectively (Figure 3C and 3D). On average the pathological cellularity estimation is about 1.7 times higher than the qpure estimation (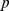-value 2.3e-13 based on a two-sample t-test).

Qpure was compared to ASCAT [5]. ASCAT estimated cellularity for only 29 of the 76 pancreatic samples (38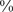) and it ranged from 34 to 64 percent (46
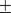
8). The correlation between ASCAT and *KRAS* estimations is 0.66. The ASCAT estimation fails to converge for 47 samples, which could be due to the low cellularity scores of those samples. The *KRAS* cellularity estimations for the 47 samples that cannot be estimated by ASCAT ranged from 7 to 51 percent (24
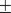
11). The pair-wise comparisons across pathology, *KRAS*, qpure and ASCAT estimates are shown in Figure S4.

## Discussion

In this study we describe a tool (qpure) for estimating tumor purity or cellularity directly from DNA samples. A key advantage of using the qpure tool for the estimation of tumor cellularity is that it is an unbiased statistical approach that directly measures tumor content from the DNA sample that will be used in downstream molecular studies. In contrast, cellularity estimates from pathology review of histology slides are based on a tissue section that may not be representative of the sample used for nucleic acid extraction.

It is known that some factors such as intra-tumor heterogeneity and tumor ploidy can confound with tumor cellularity estimation [15]. In order to mitigate the effect of these factors on our estimate of cellularity we applied a mixture model. Methods such as k-means clustering require a priori knowledge of the factors influencing the cellularity estimate; the user must pre-define the number of clusters, or factors, before the algorithm can be applied to the data. The advantage of using a mixture model is that it accounts for tumour heterogeneity and tumour ploidy information by discovering the optimal number of clusters that describe the BAF distribution in that particular sample.

The performance of the qpure model was demonstrated using three approaches: 1) the leave-one-out cross-validation analysis showed that the predictive power of the qpure model is high; 2) qpure cellularity estimates for five cell lines were all 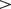 99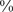; 3) qpure cellularity predictions were strongly correlated (0.87) with cellularity estimates calculated from the allele frequency of *KRAS* mutations detected by deep amplicon sequencing data within a cohort of 76 pancreatic tumours. Compared to ASCAT, qpure can predict cellularity from samples with a broad range of cellularity levels including samples with low cellularity, while ASCAT fails to converge for those samples. For samples that ASCAT could process, the qpure cellularity estimates were more similar to *KRAS* estimates than ASCAT estimates. The correlation of cellularity estimates by pathology and qpure within the cohort of primary pancreatic tumours was low. This is likely because the pathology analysis is done on a 2-dimensional section of the tissue that may not reflect the cellularity of the sample used for nucleic acid extraction and genomic studies. These results suggest that qpure could be a useful tool for estimating tumor cellularity with high accuracy and low error rate.

A limitation of the qpure method is that currently it is based on Illumina genome-wide SNP data, however, qpure does not depend on the resolution of the SNP array used. The model can also be applied to other chips such as HumanOmni2.5 and HumanOmni5-Quad. As long as the B Allele Frequency and log R ratio values are provided, the tumour cellularity of the samples can be estimated. Another requirement of the qpure method is that the paired tumour-normal SNP data sets are used in the analysis so that heterozygous SNPs in the normal sample can be selected.

qpure is an effective method for estimating tumour cellularity in samples to be used for cancer genomic studies where the presence of normal tissue in the tumor sample can significantly affect downstream analyses. The qpure method has been implemented in an R package and can be downloaded from https://sourceforge.net/projects/qpure/.

## Supporting Information

Figure S1(A) The number of normal het SNP array probes on LOH regions in the mixture experiment. (B) The distribution of BAF for the normal het SNPs on LOH regions in the mixture experiment. Among 260257 heterozygous SNP probes in the normal tissue, qpure looks for those that are in regions of LOH in the tumour. In the mixture experiment the number of SNP array probes was 12810, 18406, 17633, 16413, 16671, 16492, 17324, 12994, 13717, 12954, 18545, 11216, 12186 and 13004 for 100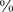 down to 0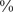 respectively. Number of probes might vary in each mixture due the threshold method used. SNP probes are identified by qpure as present in regions of loss at 85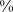, 80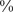, 75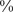, 65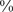, 60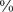, 50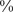, 40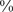, 30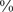, 20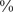, 15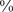, 10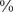, 5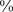 and 0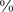 tumour DNA (A). The distribution of these SNP array probes for each mixture is shown (B).(PDF)

Figure S2 **Prediction model of tumor cellularity using d-score in the mixing experiment.** (A) fit simple linear regression model with mixture clustering (B) fit spline regression model with mixture clustering (C) fit simple linear regression model with k-means clustering (D) fit spline regression model with k-means clustering. In the plots the solid line is the fitted model and the dash lines are its prediction intervals. The tables showed the estimates of main parameters used in each model and the adjusted R-squared.
(PDF)

Figure S3
**D-score estimates using different thresholds to select probes in LOH regions for samples with different percentage of tumor DNA.** The amount of tumor DNA in the samples decreased from the left to the right. The “mycutoff” value is equal to the median of all the selected SNPs minus the standard deviation of middle 50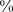 quantile. The figure showed that the change of cutoff value for the selection of probes do not affect the d-score.(PDF)

Figure S4 **Pair-wise correlations between cellularity estimates across four different methods: pathology, qpure, KRAS sequencing and ASCAT for the 76 pancreatic tumour samples.** As the pair-wise correlaitons get bigger the font size gets bigger. The red line in the scatter plot showed a linear correlation between each pair of the estimates.
(PDF)

Table S1 (PDF)

Table S2 (PDF)

Table S3 (PDF)

Text S1 (PDF)
