# Supplementary figures and images for: qpure: A Tool to Estimate Tumor Cellularity from Genome-Wide Single-Nucleotide Polymorphism Profiles
Source: PLoS One. 2012 Sep 25;7(9):e45835. doi: 10.1371/journal.pone.0045835 (PMC3457972; doi:10.1371/journal.pone.0045835)

(A) Numbers of normal het SNPs on LOH regions      (B) The distribution of BAF for LOH regions

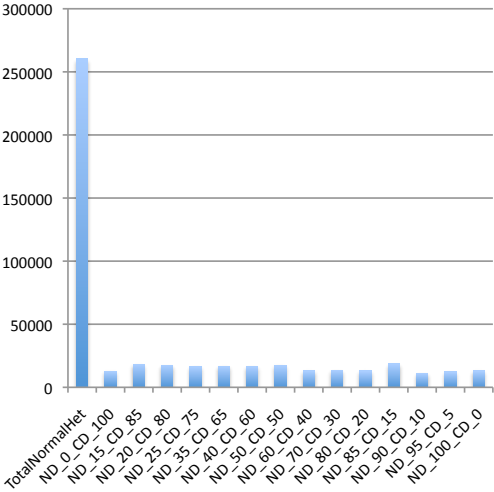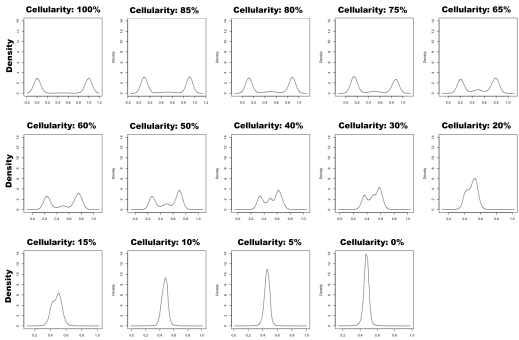

Supplement: Figure S1 — (A) The number of normal het SNP array probes on LOH regions in the mixture experiment. (B) The distribution of BAF for the normal het SNPs on LOH regions in the mixture experiment. Among 260257 heterozygous SNP probes in the normal tissue, qpure looks for those that are in regions of LOH in the tumour. In the mixture experiment the number of SNP array probes was 12810, 18406, 17633, 16413, 16671, 16492, 17324, 12994, 13717, 12954, 18545, 11216, 12186 and 13004 for 100 down to 0 respectively. Number of probes might vary in each mixture due the threshold method used. SNP probes are identified by qpure as present in regions of loss at 85, 80, 75, 65, 60, 50, 40, 30, 20, 15, 10, 5 and 0 tumour DNA (A). The distribution of these SNP array probes for each mixture is shown (B). (PDF) [file pone.0045835.s001.pdf]

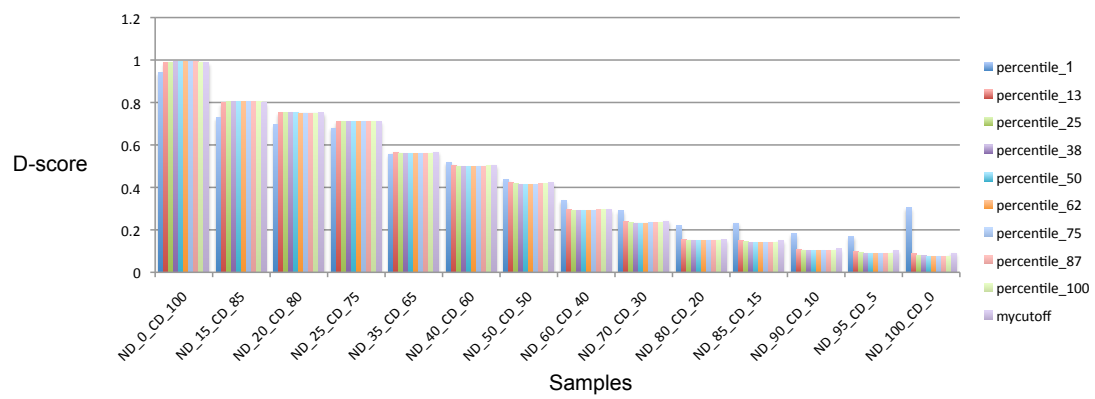

Supplement: Figure S3 — D-score estimates using different thresholds to select probes in LOH regions for samples with different percentage of tumor DNA. The amount of tumor DNA in the samples decreased from the left to the right. The “mycutoff” value is equal to the median of all the selected SNPs minus the standard deviation of middle 50 quantile. The figure showed that the change of cutoff value for the selection of probes do not affect the d-score. (PDF) [file pone.0045835.s003.pdf]

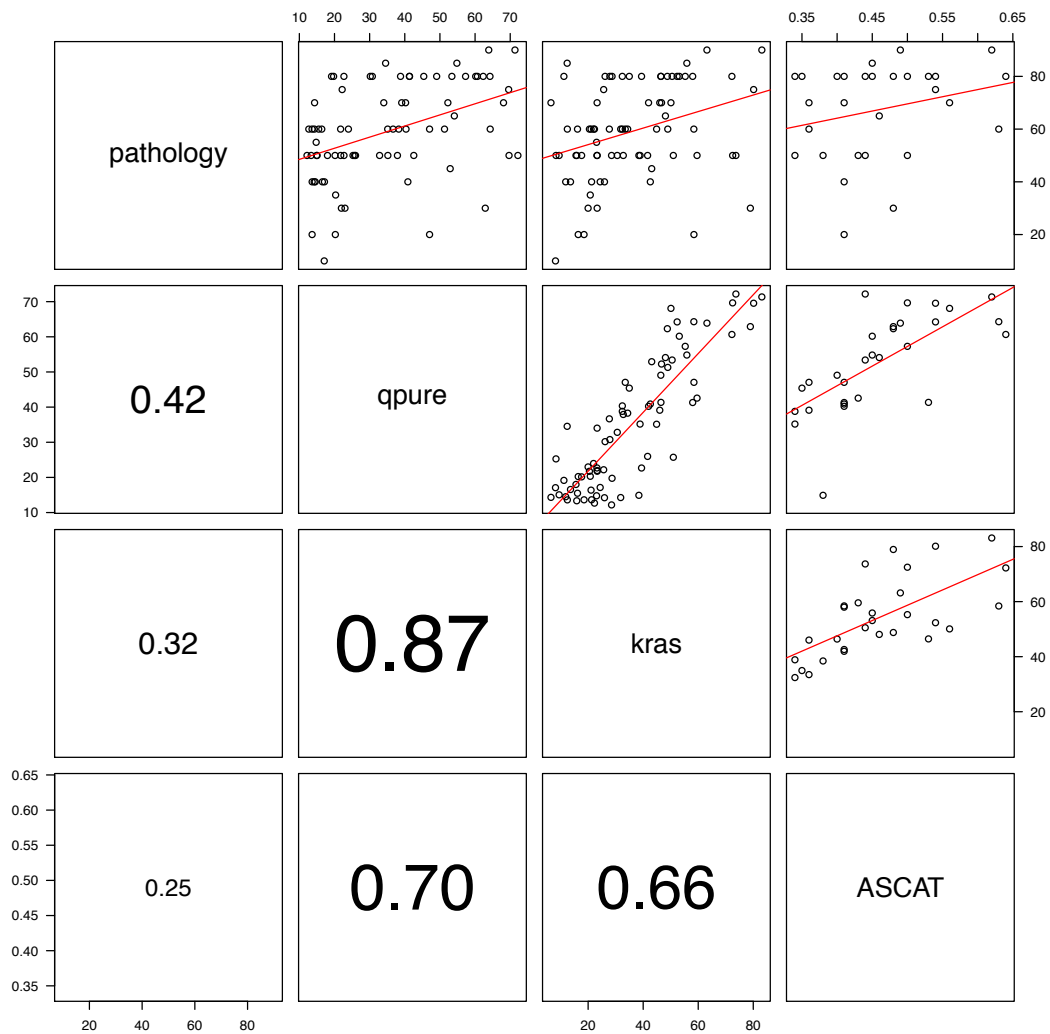

Supplement: Figure S4 — Pair-wise correlations between cellularity estimates across four different methods: pathology, qpure, KRAS sequencing and ASCAT for the 76 pancreatic tumour samples. As the pair-wise correlaitons get bigger the font size gets bigger. The red line in the scatter plot showed a linear correlation between each pair of the estimates. (PDF) [file pone.0045835.s004.pdf]
