# Supplementary material for: qpure: A Tool to Estimate Tumor Cellularity from Genome-Wide Single-Nucleotide Polymorphism Profiles
Source: PLoS One. 2012 Sep 25;7(9):e45835. doi: 10.1371/journal.pone.0045835 (PMC3457972; doi:10.1371/journal.pone.0045835)

Mixture clustering model

	Linear (A)	Spline (B)
Intercept P-value	1.23E-06	3.01E-14
Predictor P-value	2.65E-11	1.85E-11
Adjusted R-squared	99.65%	99.20%

K-means clustering model

	Linear (C)	Spline (D)
Intercept P-value	0.001	2.72E-13
Predictor P-value	1.74E-09	2.68E-10
Adjusted R-squared	99%	99.6%

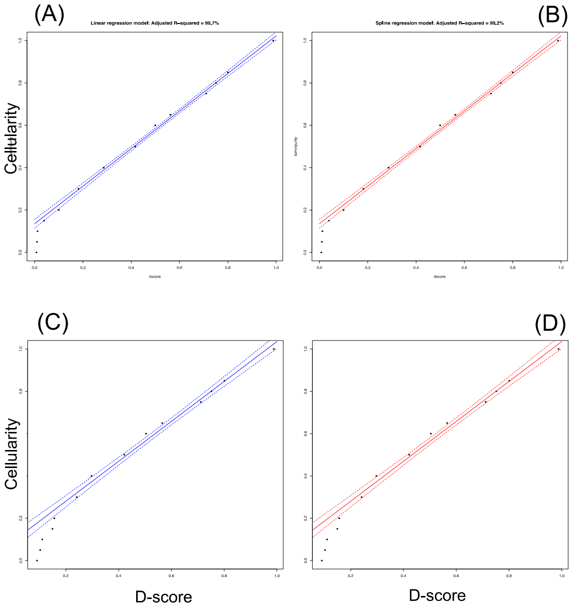

Supplement: Figure S2 — Prediction model of tumor cellularity using d-score in the mixing experiment. (A) fit simple linear regression model with mixture clustering (B) fit spline regression model with mixture clustering (C) fit simple linear regression model with k-means clustering (D) fit spline regression model with k-means clustering. In the plots the solid line is the fitted model and the dash lines are its prediction intervals. The tables showed the estimates of main parameters used in each model and the adjusted R-squared. (PDF) [file pone.0045835.s002.pdf]
