# Supplementary material for: qpure: A Tool to Estimate Tumor Cellularity from Genome-Wide Single-Nucleotide Polymorphism Profiles
Source: PLoS One. 2012 Sep 25;7(9):e45835. doi: 10.1371/journal.pone.0045835 (PMC3457972; doi:10.1371/journal.pone.0045835)
Supplement: Table S1 — (PDF) [file pone.0045835.s005.pdf]

| SampleID | Cellularity estimates | | | | qpure | | Source |
|---|---|---|---|---|---|---|---|
| | pathology | qpure | kras | ascat | peaks | dscore | |
| ICGC_0002 | 85 | 54.82 | 55.87 | 45 | 4 | 0.289 | APGI |
| ICGC_0003 | 50 | 25.27 | 8.32 | - | 4 | 0.073 | APGI |
| ICGC_0005 | 50 | 25.73 | 50.96 | - | 5 | 0.076 | APGI |
| ICGC_0007 | 80 | 53.40 | 50.52 | 44 | 6 | 0.276 | APGI |
| ICGC_0008 | 10 | 17.08 | 8.14 | - | 3 | 0.028 | APGI |
| ICGC_0009 | 90 | 71.35 | 83.12 | 62 | 4 | 0.472 | APGI |
| ICGC_0010 | 70 | 39.12 | 46.03 | 36 | 4 | 0.162 | APGI |
| ICGC_0011 | 50 | 37.91 | 32.78 | - | 5 | 0.153 | APGI |
| ICGC_0012 | 80 | 41.35 | 46.46 | 53 | 5 | 0.178 | APGI |
| ICGC_0013 | 80 | 22.68 | 39.41 | - | 3 | 0.058 | APGI |
| ICGC_0014 | 50 | 20.16 | 17.63 | - | 3 | 0.044 | APGI |
| ICCG_0015 | 80 | 19.75 | 28.69 | - | 3 | 0.042 | APGI |
| ICGC_0016 | 60 | 51.30 | 48.94 | - | 6 | 0.258 | APGI |
| ICGC_0017 | 50 | 18.00 | 15.59 | - | 4 | 0.032 | APGI |
| ICGC_0019 | 30 | 22.98 | 19.99 | - | 3 | 0.060 | APGI |
| ICGC_0077 | 40 | 16.56 | 13.60 | - | 3 | 0.025 | APGI |
| ICGC_0020 | 35 | 20.32 | 20.78 | - | 3 | 0.045 | APGI |
| ICGC_0021 | 20 | 47.05 | 58.44 | 41 | 4 | 0.222 | APGI |
| ICGC_0022 | 50 | 25.98 | 41.61 | - | 3 | 0.077 | APGI |
| ICGC_0023 | 30 | 21.96 | 23.28 | - | 3 | 0.054 | APGI |
| ICGC_0024 | 80 | 19.16 | 11.20 | - | 3 | 0.039 | APGI |
| ICGC_0078 | 20 | 13.63 | 18.51 | - | 3 | 0.010 | APGI |
| ICGC_0027 | 40 | 17.14 | 24.38 | - | 3 | 0.028 | APGI |
| ICGC_0028 | 85 | 34.54 | 12.38 | - | 4 | 0.130 | APGI |
| ICGC_0079 | 40 | 14.51 | 11.85 | - | 3 | 0.014 | APGI |
| ICGC_0030 | 50 | 21.76 | 23.29 | - | 3 | 0.053 | APGI |
| ICGC_0031 | 80 | 45.40 | 34.96 | 35 | 5 | 0.209 | APGI |
| ICGC_0032 | 50 | 35.18 | 38.87 | 34 | 6 | 0.135 | APGI |
| ICGC_0033 | 60 | 47.05 | 33.49 | 36 | 3 | 0.222 | APGI |
| ICGC_0034 | 60 | 38.28 | 34.37 | - | 5 | 0.156 | APGI |
| ICGC_0035 | 45 | 52.92 | 43.10 | - | 5 | 0.272 | APGI |
| ICGC_0036 | 50 | 72.18 | 73.69 | 44 | 7 | 0.483 | APGI |
| ICGC_0037 | 50 | 69.66 | 72.50 | 50 | 6 | 0.451 | APGI |
| ICGC_0038 | 70 | 14.33 | 6.50 | - | 3 | 0.013 | APGI |
| ICGC_0039 | 80 | 41.31 | 57.99 | 41 | 4 | 0.178 | APGI |
| ICGC_0040 | 40 | 40.88 | 42.60 | 41 | 4 | 0.175 | APGI |
| ICGC_0041 | 60 | 14.27 | 31.83 | - | 3 | 0.013 | APGI |
| ICGC_0042 | 60 | 35.13 | 44.88 | - | 5 | 0.134 | APGI |
| ICGC_0043 | 80 | 30.77 | 27.90 | - | 5 | 0.106 | APGI |
| ICGC_0044 | 60 | 21.74 | 20.47 | - | 4 | 0.053 | APGI |
| ICGC_0045 | 80 | 38.79 | 32.42 | 34 | 9 | 0.159 | APGI |
| ICGC_0088 | 30 | 62.90 | 78.95 | 48 | 4 | 0.371 | APGI |
| ICGC_0046 | 80 | 30.18 | 26.16 | - | 4 | 0.102 | APGI |
| ICGC_0047 | 50 | 15.05 | 9.46 | - | 3 | 0.017 | APGI |
| ICGC_0048 | 80 | 62.31 | 48.78 | 48 | 4 | 0.365 | APGI |
| ICGC_0090 | 50 | 13.36 | 15.86 | - | 3 | 0.008 | APGI |
| ICGC_0049 | 50 | 22.70 | 23.20 | - | 3 | 0.058 | APGI |
| ICGC_0050 | 50 | 12.18 | 28.51 | - | 3 | 0.002 | APGI |
| ICGC_0052 | 70 | 52.26 | 46.67 | - | 5 | 0.266 | APGI |
| ICGC_0092 | 40 | 13.64 | 21.26 | - | 3 | 0.010 | APGI |
| ICGC_0093 | 70 | 40.23 | 41.96 | 41 | 3 | 0.170 | APGI |

| | | | | | | | |
|---|---|---|---|---|---|---|---|
| ICGC_0096 | 60 | 23.96 | 21.97 | - | 3 | 0.065 | APGI |
| ICGC_0054 | 80 | 49.07 | 46.41 | 40 | 3 | 0.239 | APGI |
| ICGC_0055 | 80 | 60.17 | 53.13 | 45 | 6 | 0.342 | APGI |
| ICGC_0097 | 20 | 20.24 | 16.40 | - | 3 | 0.044 | APGI |
| ICGC_0056 | 50 | 14.91 | 38.44 | 38 | 2 | 0.016 | APGI |
| ICGC_0057 | 60 | 40.35 | 32.43 | - | 8 | 0.171 | APGI |
| ICGC_0058 | 60 | 13.61 | 12.45 | - | 3 | 0.010 | APGI |
| ICGC_0060 | 70 | 34.02 | 23.26 | - | 4 | 0.127 | APGI |
| ICGC_0061 | 90 | 63.89 | 63.17 | 49 | 6 | 0.382 | APGI |
| ICGC_0099 | 40 | 14.22 | 25.94 | - | 3 | 0.013 | APGI |
| ICGC_0062 | 75 | 22.18 | 25.64 | - | 4 | 0.055 | APGI |
| ICGC_0101 | 55 | 14.78 | 23.07 | - | 3 | 0.016 | APGI |
| ICGC_0102 | 60 | 16.38 | 21.06 | - | 4 | 0.024 | APGI |
| ICGC_0204 | 80 | 60.67 | 72.24 | 64 | 5 | 0.347 | APGI |
| ICGC_0104 | 60 | 15.50 | 16.14 | - | 3 | 0.019 | APGI |
| ICGC_0109 | 65 | 54.10 | 48.09 | 46 | 7 | 0.283 | APGI |
| ICGC_0064 | 60 | 36.67 | 27.71 | - | 3 | 0.145 | APGI |
| ICGC_0205 | 70 | 68.09 | 50.09 | 56 | 6 | 0.431 | APGI |
| ICGC_0206 | 75 | 69.55 | 80.13 | 54 | 4 | 0.449 | APGI |
| ICGC_0207 | 60 | 64.30 | 58.41 | 63 | 5 | 0.387 | APGI |
| ICGC_0208 | 60 | 12.69 | 22.30 | - | 2 | 0.005 | APGI |
| ICGC_0115 | 80 | 57.29 | 55.28 | 50 | 3 | 0.313 | APGI |
| ICGC_0066 | 80 | 64.24 | 52.34 | 54 | 6 | 0.386 | APGI |
| ICGC_0118 | 50 | 42.56 | 59.57 | 43 | 3 | 0.187 | APGI |
| ICGC_0067 | 50 | 32.83 | 30.59 | - | 4 | 0.119 | APGI |
| ICGC_0005CD | - | 99.85 | - | 96 | 3 | 0.983 | APGI cell line |
| ICGC_0008CD | - | 100.00 | - | 94 | 3 | 0.995 | APGI cell line |
| ICGC_0077CD | - | 99.54 | - | 96 | 6 | 0.976 | APGI cell line |
| ICGC_0036CD | - | 100.00 | - | 99 | 3 | 0.995 | APGI cell line |
| ICGC_0054CD | - | 99.99 | - | 97 | 4 | 0.986 | APGI cell line |

Table S1: Sample cellularity estimations from pathology, qpure, *KRAS sequencing and ascat* for a total of 76 pancreatic tumour samples and 5 pancreatic cell lines. Both the number of optimal clusters (peaks) estimated by the mixture model and d-scores (dscore) were generated by the qpure method only.
