# Supplementary material for: qpure: A Tool to Estimate Tumor Cellularity from Genome-Wide Single-Nucleotide Polymorphism Profiles
Source: PLoS One. 2012 Sep 25;7(9):e45835. doi: 10.1371/journal.pone.0045835 (PMC3457972; doi:10.1371/journal.pone.0045835)
Supplement: Table S2 — (PDF) [file pone.0045835.s006.pdf]

Exon	Codon	Left primer (including torrent adaptors)	Right primer (including torrent adaptors)
2	4-14	CCATCTCATCCCTGCGTGTCTCCGACTCAG_10bp_barcode_GCCTGCTGAAAATGACTGAA	CCTCTCTATGGGCAGTCGGTGATTATCGTCAAGGCACTCTTGC
3	55-63	CCATCTCATCCCTGCGTGTCTCCGACTCAG_10bp_barcode_ACTGGTCCCTCATTGCACTGT	CCTCTCTATGGGCAGTCGGTGATTGATGGAGAAACCTGTCTCTTGGA

Table S2: Primers used for Ion Torrent amplicon sequencing of *KRAS* mutations
