# Supplementary material for: qpure: A Tool to Estimate Tumor Cellularity from Genome-Wide Single-Nucleotide Polymorphism Profiles
Source: PLoS One. 2012 Sep 25;7(9):e45835. doi: 10.1371/journal.pone.0045835 (PMC3457972; doi:10.1371/journal.pone.0045835)
Supplement: Table S3 — (PDF) [file pone.0045835.s007.pdf]

SampleID	Chr12 Position	Base Change	Number of Wildtype Reads	Number of Mutant Reads	KRAS cellularity
ICGC_0002	25398284	C>T	4623	1792	55.87
ICGC_0003	25380275; 25398284	T>G; C>T	19281; 18637	614; 1030	8.32
ICGC_0005	25398284	C>A	6338	2167	50.96
ICGC_0007	25398284	C>T	6246	2111	50.52
ICGC_0008	25398285	C>G	8227	349	8.14
ICGC_0009	25398284	C>T	5311	3777	83.12
ICGC_0010	25398285	C>G	3014	901	46.03
ICGC_0011	25380276	T>C	10457	2050	32.78
ICGC_0012	25398284	C>A	3582	1084	46.46
ICGC_0013	25398284	C>A	10370	2545	39.41
ICGC_0014	25398284	C>A	6641	642	17.63
ICCG_0015	25398284	C>T	5958	998	28.69
ICGC_0016	25398284	C>A	11582	3752	48.94
ICGC_0017	25398284	C>A	3419	289	15.59
ICGC_0019	25398285	C>G	3467	385	19.99
ICGC_0077	25398284	C>A	18001	1313	13.6
ICGC_0020	25398284	C>T	12202	1415	20.78
ICGC_0021	25398284	C>T	3207	1324	58.44
ICGC_0022	25398284	C>T	17244	4530	41.61
ICGC_0023	25398284	C>T	3933	518	23.28
ICGC_0024	25398284	C>T	7633	453	11.2
ICGC_0078	25398284	C>T	1686	172	18.51
ICGC_0027	25398284	C>A	1664	231	24.38
ICGC_0028	25398284	C>T	2107	139	12.38
ICGC_0079	25398284	C>A	1524	96	11.85
ICGC_0030	25380275	T>G	9402	1239	23.29
ICGC_0031	25398284	C>A	4291	909	34.96
ICGC_0032	25398284	C>A	3838	926	38.87
ICGC_0033	25398284	C>T	14209	2858	33.49
ICGC_0034	25398284	C>T	9483	1968	34.37
ICGC_0035	25398284	C>T	3185	875	43.1
ICGC_0036	25398284	C>T	4863	2837	73.69
ICGC_0037	25398285	C>G	1479	841	72.5
ICGC_0038	25398284	C>T	4945	166	6.5
ICGC_0039	25398284	C>T	1479	604	57.99
ICGC_0040	25398284	C>A	3203	867	42.6
ICGC_0041	25398284	C>T	4015	760	31.83
ICGC_0042	25398285	C>G	11826	3422	44.88
ICGC_0043	25398284	C>A	1172	190	27.9
ICGC_0044	25380276	T>C	9305	1061	20.47
ICGC_0045	25398285	C>G	2176	421	32.42
ICGC_0088	25398284	C>T	1432	934	78.95
ICGC_0046	25380275	T>G	3150	474	26.16
ICGC_0047	25398284	C>T	5134	255	9.46
ICGC_0048	25398283; 25398284	A>C; C>A	5995; 5687	1882; 1884	48.78
ICGC_0090	25398285	C>A	1718	148	15.86
ICGC_0049	25398284	C>A	4313	566	23.2

ICGC_0050	25398284	C>T	1522	253	28.51
ICGC_0052	25398284	C>T	1242	378	46.67
ICGC_0092	25398285	C>G	1152	137	21.26
ICGC_0093	25398284	C>T	1695	450	41.96
ICGC_0096	25398284	C>T	794	98	21.97
ICGC_0054	25398284	C>A	738	223	46.41
ICGC_0055	25398285	C>G	915	331	53.13
ICGC_0097	25398285	C>G	795	71	16.4
ICGC_0056	25398285	C>G	1286	306	38.44
ICGC_0057	25398284	C>A	1111	215	32.43
ICGC_0058	25398284	C>A	3270	217	12.45
ICGC_0060	25398285	C>G	1824	240	23.26
ICGC_0061	25398284	C>A	795	367	63.17
ICGC_0099	25398285	C>G	1704	254	25.94
ICGC_0062	25398284	C>A	782	115	25.64
ICGC_0101	25398284	C>T	698	91	23.07
ICGC_0102	25398284	C>A	1283	151	21.06
ICGC_0204	25398284	C>T	2545	1439	72.24
ICGC_0104	25398284	C>A	1526	134	16.14
ICGC_0109	25398284	C>A	1355	429	48.09
ICGC_0064	25398284; 25398285	C>A; C>G	1284; 1309	220; 197	27.71
ICGC_0205	25380275	T>G	2819	942	50.09
ICGC_0206	25398284	C>T	365	244	80.13
ICGC_0207	25398284	C>T	892	368	58.41
ICGC_0208	25398285	C>G	1315	165	22.3
ICGC_0115	25398285	C>G	877	335	55.28
ICGC_0066	25398284	C>T	505	179	52.34
ICGC_0118	25380275	T>G	2876	1220	59.57
ICGC_0067	25380277; 25380278	G>T;A>T	2614; 2610	472; 479	30.59

Table S3: Cellularity estimations of 76 pancreatic tumour samples using deep Ion Torrent sequencing of *KRAS* mutations.
