# Supplementary material for: qpure: A Tool to Estimate Tumor Cellularity from Genome-Wide Single-Nucleotide Polymorphism Profiles
Source: PLoS One. 2012 Sep 25;7(9):e45835. doi: 10.1371/journal.pone.0045835 (PMC3457972; doi:10.1371/journal.pone.0045835)
Supplement: Text S1 — (PDF) [file pone.0045835.s008.pdf]

AUSTRALIAN PANCREATIC CANCER GENOME INITIATIVE (APGI),

A MEMBER OF THE INTERNATIONAL CANCER GENOME CONSORTIUM

GARVAN INSTITUTE OF MEDICAL RESEARCH
Sydney, Australia

Andrew V. Biankin
Amber L. Johns
Amanda Mawson
David K. Chang
Mary-Anne L. Brancato
Sarah J. Rowe
Skye L. Simpson
Lorraine A. Chantrill
Venessa T. Chin
Angela Chou
Mark J. Cowley
Jeremy L. Humphris
Marc D. Jones
R. Scott Mead
Adnan M. Nagrial
Marina Pajic
Jessica Pettit
Mark Pinese
Ilse Rooman
Jianmin Wu
Roger J. Daly
Elizabeth A. Musgrove
Robert L. Sutherland

Queensland Center for Medical Genomics, Institute for Molecular Bioscience
University of Queensland, St Lucia, Queensland

Sean M. Grimmond
Nicola Waddell
Karin S Kassahn
David K. Miller
Peter J. Wilson
Ann-Marie Patch
Sarah Song
Ivon Harliwong
Senel Idrisoglu
Craig Nourse
Ehsan Nourbakhsh
Suzanne Manning
Shivangi Wani
Milena Gongora

Matthew Anderson
Oliver Holmes
Conrad Leonard
Darrin Taylor
Scott Wood
Christina Xu
Katia Nones
J. Lynn Fink
Angelika Christ
Tim Bruxner
Nicole Cloonan
Felicity Newell
John V. Pearson

ROYAL NORTH SHORE HOSPITAL
St Leonards, New South Wales, Australia

Jaswinder S. Samra
Anthony J. Gill
Nick Pavlakis
Alex Guminski
Mona Martyn-Smith
Christopher Toon

BANKSTOWN HOSPITAL
Bankstown, New South Wales, Australia

Ray Asghari
Neil D. Merrett
David K. Chang
Darren A. Pavey
Amithabad Das

LIVERPOOL HOSPITAL
Liverpool, New South Wales, Australia

Peter H. Cosman
Kasim Ismail
Chelsie O'Connor

WESTMEAD HOSPITAL
Westmead, New South Wales, Australia

Vincent W. Lam
Duncan McLeod
Henry C. Pleass

Virginia James

ROYAL PRINCE ALFRED HOSPITAL
Camperdown, New South Wales, Australia

James G. Kench
Caroline L. Cooper
David Joseph
Charbel Sandroussi

FREMANTLE HOSPITAL
Fremantle, New South Wales, Australia

Michael Texler
Cindy Forrest
Andrew Laycock
Krishna P. Epari
Mo Ballal
David R. Fletcher
Sanjay Mukhedkar

SIR CHARLES GAIRDNER HOSPITAL
Nedlands, Western Australia, Australia

Nigel A. Spry
Bastiaan DeBoer
Ming Chai
Kynan Feeney

ST JOHN OF GOD HEALTHCARE
Subiaco, Western Australia, Australia

Nikolajs Zeps
Maria Beilin

ROYAL ADELAIDE HOSPITAL
Adelaide, South Australia, Australia

Nam Q. Nguyen
Andrew R. Ruszkiewicz
Chris Worthley
Chuan P. Tan
Tamara Debrencini

FLINDERS MEDICAL CENTER
Bedford Park, South Australia, Australia

John Chen
Mark E. Brooke-Smith
Virginia Papangelis

GREENSLOPES PRIVATE HOSPITAL
Greenslopes, Queensland, Australia

Henry Tang
Andrew P. Barbour

ENVOI PATHOLOGY
Herston, Queensland

Andrew D. Clouston
Patrick Martin

PRINCESS ALEXANDA HOSPITAL
Woolloongabba, Queensland, Australia

Thomas J. O'Rourke
Amy Chiang
Jonathan W. Fawcett
Kellee Slater
Shinn Yeung
Michael Hatzifotis
Peter Hodgkinson

AUSTIN HOSPITAL
Heidelberg, Victoria, Australia

Christopher Christophi
Mehrdad Nikfarjam
Victorian Cancer Biobank

International Collaborators

Johns Hopkins Medical Institutes
Baltimore, USA

James R. Eshleman
Ralph H. Hruban
Anirban Maitra
Christine A. Iacobuzio-Donahue
Richard D. Schulick
Christopher L. Wolfgang
Richard A. Morgan

ARC-NET CENTER FOR APPLIED RESEARCH ON CANCER, UNIVERSITY AND HOSPITAL TRUST OF VERONA
Verona, Italy

Rita T. Lawlor
Stefania Beghelli
Vincenzo Corbo
Maria Scardoni
Claudio Bassi

University of California,
San Francisco USA

Margaret A. Tempero
